# A Case Report of Congenital Fiber Type Disproportion with an Increased Level of Anti-ACh Receptor Antibodies

**DOI:** 10.1155/2013/607678

**Published:** 2013-05-16

**Authors:** Shigemi Kimura, Shiro Ozasa, Keiko Nomura, Hirofumi Kosuge, Kowasi Yoshioka

**Affiliations:** Department of Child Development, Kumamoto University Graduate School, 1-1-1 Honjo, Kumamoto 860-0811, Japan

## Abstract

Congenital fiber type disproportion (CFTD) is a form of congenital myopathy, which is defined by type 1 myofibers that are 12% smaller than type 2 myofibers, as well as a general predominance of type 1 myofibers. Conversely, myasthenia gravis (MG) is an acquired immune-mediated disease, in which the acetylcholine receptor (AChR) of the neuromuscular junction is blocked by antibodies. Thus, the anti-AChR antibody is nearly specific to MG. Herein, we report on a case of CFTD with increased anti-AChR antibody levels. A 23-month-old boy exhibited muscle hypotonia and weakness. Although he could walk by himself, he easily fell down and could not control his head for a long time. His blood test was positive for the anti-AChR antibody, while a muscle biopsy revealed characteristics of CFTD. We could not explain the relationship between MG and CFTD. However, we considered different diagnoses aside from MG, even when the patient's blood is positive for the anti-AChR antibody.

## 1. Introduction

Congenital fiber type disproportion (CFTD) is a form of congenital myopathy [1]. CFTD is defined as a type 1 myofiber that is 12% smaller than the type 2 myofiber. Fiber type 1 predominance, where type 1 fibers can occupy more than 55% of all fiber types, has been seen in many cases. CFTD is usually characterized by hypotonia and mild-to-severe generalized muscle weakness at birth or within the first year of life. CFTD is often associated with a high-arched palate, kyphoscoliosis, contracture, and, less commonly, a mild increase in CK levels. Mutations of actin alpha 1 skeletal muscle (ACTA1), and several genes [2–5] have all been associated with CFTD. Fiber type disproportion is a morphological finding common to cases of neurogenic atrophy and many other congenital myopathies, such as nemaline myopathy (NM) and centronuclear myopathy (CNM). CFTD requires diagnosis by exclusion of nemaline and other myopathies.

Myasthenia gravis (MG) is an acquired immune-mediated disease, in which the acetylcholine receptor of the neuromuscular junction is blocked by antibodies [6]. The disease is roughly classified into generalized and ocular myasthenia gravis (GMG and OMG, resp.). The symptoms of GMG involve easy fatigability of the skeletal or bulbar muscles, which results in dysphonia, dysphagia, general fatigue, and occasionally respiratory failure. The predominant symptoms of OMG are extraocular muscle weakness, ptosis, and limitations of eye movements. Daily variation in symptoms, with a worsening of muscle weakness in the evening, is a characteristic finding of OMG. The diagnosis of MG conditions is established by the history, physical examination, and laboratory data, including a Tensilon test, anti-AChR antibody titers, and electromyogram (EMG). Therapeutic options for MG include anticholinesterases, corticosteroids, immune suppressive agents, thymectomy, and plasmapheresis. A positive finding with the anti-AhR antibody indicates specificity to MG [7].

Herein, we report on the first case of CFTD with an increased level of anti-AChR antibodies.

## 2. Case Report

The male patient was born after 38 weeks and 3 days of gestation with a birth weight of 2350 g. Although the patient had a twin in utero, the sibling died before birth. The patient had a short stature and failed to gain weight. He could walk by himself at an age of 11 months, without the developmental step of crawling.

At the last examination, the patient was 23 months old, with a body height of 77.7 cm (−2.1SD) and a weight of 8.4 kg (−2.2SD). He easily fell down and had hypotonia and muscle weakness of the whole body. Muscle weakness of the neck was very apparent, with the patient having great difficulty supporting his head for a long time. The severity of muscle weakness did not show a daily variation. The patient did not have ptosis, opthalmoplegia, a nasally voice, or difficulty in biting hard on food but did present with a high-arched palate. The Tensilon test showed the muscle weakness was not changed. His brain MRI, chest X-ray, and nerve conduction velocity were normal. Additionally, the EMG did not reveal a neurogenic and myogenic pattern, and an evoked EMG did not show waning, a common finding among MG cases. His levels of creatine kinase (80 U/l; normal range, 67–284) were normal. His levels of anti-AChR antibodies were high (1.0 nmol/L; normal range, <0.1), while his levels of antimuscle-specific tyrosine kinase (anti-MuSK) antibodies were normal (0.005 nmol/L; normal range, <0.05). The anti-AChR antibody was reexamined and remained high. Thus, due to the increased levels of anti-AChR antibodies, we made a diagnosis of GMG.

The patient was administered 5 mg/kg of pyridostigmine every day and 2 mg/kg of oral prednisolone every other day. However, the treatment led to no response. Subsequently, 3 courses of steroid pulse therapy (30 mg/kg/day × 3 days/1 week per course) were performed but also resulted in no response. During the pulse therapy, anti-AChR antibody changed from positive to negative.

**Figure 1:**
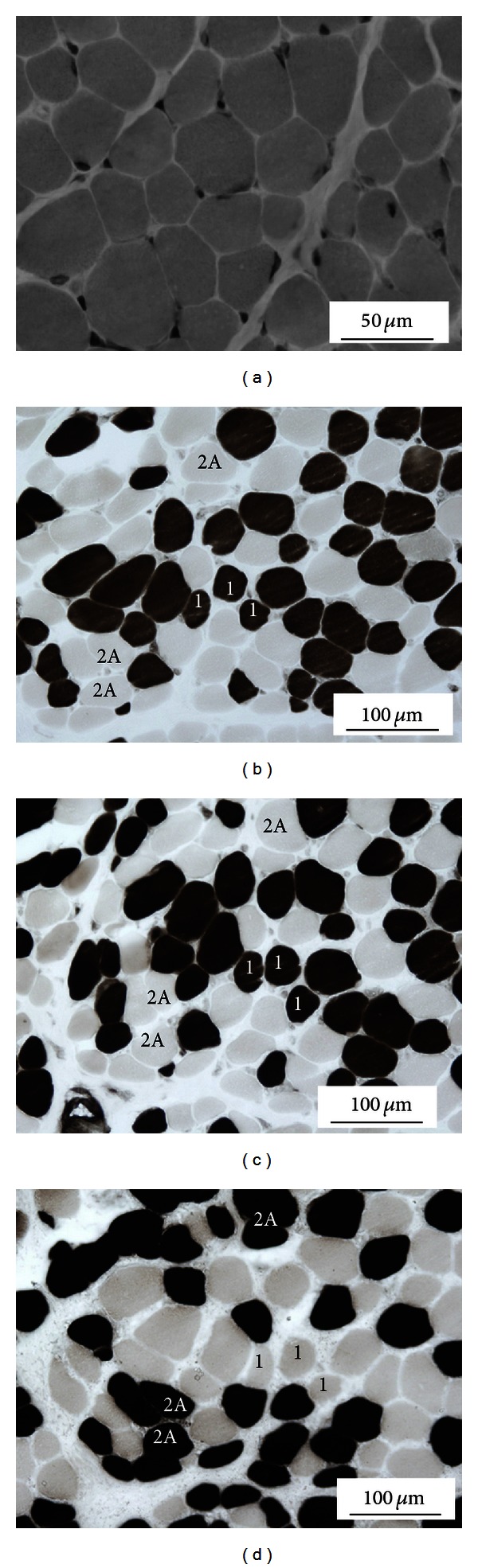
Cryostat sections of the patient's muscle biopsy. (a) Hematoxylin and eosin staining reveals clear variations in myofiber size. (b), (c), and (d) ATPase staining at 4.2 (b), 4.6 (c), and 11 (d). The “1” and “2A” in the figure denote type 1 and 2A fibers, respectively. The findings indicate that type 1 fibers were 12% smaller than type 2 fibers, while type 2B fibers were deficient.

A muscle biopsy was performed to decide a proper diagnosis. Hematoxylin and eosin stains showed clear variations in myofiber size (Figure 1(a)). ATPase stain revealed type 1 fibers that were 12% smaller than type 2 fibers (Figure 1(b)). However, fiber type 1 predominance was not observed, as type 1 fiber accounted for 50.8% of all fibers (Figures 1(b), 1(c), and 1(d)). However, the proportion of the type 1 fiber showed a trend for being higher, because the ratio of type 1 to 2 myofibers was 1 to 2. As illustrated in Figure 1(c), there was a deficiency in type 2B fibers; a finding sometimes observed in CFTD. NADH-TR staining and other stains confirmed the absence of other congenital myopathies, such as NM, CNM, and inflammatory changes. These findings coincided with a diagnosis of CFTD.

## 3. Discussion

Somnier previously analyzed the differences in anti-AChR antibodies between healthy controls and patients with MG [7]. Using a 0.5 nmole/L, anti-AChR antibody cutoff, the finding was 99% specific to MG. Therefore, the anti-AChR antibody of our patient was clearly elevated. However, the level decreased to normal during steroid pulse therapy. The muscle weakness of the patient and elevated anti-AChR antibodies were suspected to indicate GMG. Although the patient had muscle weakness, he did not have findings typical of MG, including a waning of EMG, tensilon test, and daily variations in muscle weakness. 

The anti-AChR antibody was detected in patients with muscular disorders, facioscapulohumeral muscular dystrophy, myotonic dystrophy, and mitochondrial myopathies [8]. Some of the patients improved with immunosuppression. In addition, the positives with the anti-AChR antibody have been previously reported in Down's syndrome cases [9, 10]. Interestingly, patients with Down's syndrome also exhibit muscle hypotonia. 

On the other hand, the muscle biopsy of our patient revealed findings indicative of CFTD, although the gene mutation was not decided.

To the best of our knowledge, the relationship between CFTD and MG has not yet been reported. However, the possibility that the anti-AChR antibody induced CFTD still remains unknown.

Therefore, even if a patient is positive for the anti-AChR antibody, diseases other than MG, which also demonstrate muscle hypotonia, should be considered.
